# *STXBP1* Stop‐Loss Mutation Associated with Complex Early Onset Movement Disorder without Epilepsy

**DOI:** 10.1002/mdc3.13509

**Published:** 2022-07-23

**Authors:** Robert Spaull, Dora Steel, Katy Barwick, Prab Prabhakar, Emma Wakeling, Manju A. Kurian

**Affiliations:** ^1^ Molecular Neurosciences, Developmental Neurosciences, Zayed Centre for Research into Rare Disease in Children UCL Great Ormond Street Institute of Child Health London UK; ^2^ Department of Neurology Great Ormond Street Hospital London UK; ^3^ North East Thames Regional Genetic Service Great Ormond Street Hospital for Children NHS Foundation Trust London UK

**Keywords:** STXBP1, tremor, stop‐loss, movement disorder

*STXBP1* encodes syntaxin‐binding protein 1, a brain‐expressed membrane trafficking protein that facilitates presynaptic vesicle docking in neurotransmission. Heterozygous loss‐of‐function variants were originally associated with infantile developmental and epileptic encephalopathy (DEE4, OMIM #612164),^1^ with the phenotype later expanding to include a wide range of severe epilepsies, intellectual disability, and other neurodevelopmental disorders.^2^ Movement disorders including ataxia, tremor, dyskinesia, dystonia, bruxism and stereotypies have been described, though nearly always accompanied by epilepsy and intellectual disability.^2^

We report a child with an *STXBP1* stop‐loss variant, who presented with an infantile onset complex movement disorder without epilepsy and only mild cognitive difficulties. Now adopted, he was born to non‐consanguineous parents. His birth parents both have a history of epilepsy and learning difficulties; in addition, his father has dystonia and tremor, and paternal grandmother had dystonia. His older biological sister has learning difficulties, joint hypermobility and attention deficit hyperactivity disorder (ADHD) but no movement disorder or epilepsy. Following a normal pregnancy and birth, tremor was evident from 6 weeks of age, rhythmic rocking movements from 6 months, and his development was delayed; he walked independently at 4 years and first words were noted from 2 years of age. He has writing difficulties and frequent falls which cause injury. His tremor is worsened by anxiety, fatigue or heightened emotion and later in the day his posture becomes crouched. His sleep pattern is disrupted with lower limb night cramps. He attends a mainstream school where he has extra support including speech and language therapy. He has additional diagnoses of ADHD, autistic spectrum disorder and hypermobile Ehlers‐Danlos syndrome. Trials of Levodopa, Trihexyphenidyl and Levetiracetam had no perceived benefit and were discontinued. Melatonin has been effective in improving sleep. Examination at age 9 years revealed marked action‐induced tremor of both arms and hands and intermittent dystonic finger posturing, tremulous tongue movements and difficulty on tandem walking (Video 1).

**Video 1:** *First segment*: In this video when he is 5 years old, fine motor action elicits tremulous finger movements and intermittent dystonic finger posturing. Age 6, when learning to ride a scooter, he demonstrates reasonable balance and fluidity to pushing without falling. *Second segment*: During assessment age 9, he has mild tremor at rest but marked distal limb tremor seen with arms held in midline or out in front, as well as intermittent dystonic finger posturing, and added distal hyperkinetic movements of larger amplitude, some which are possibly myoclonic in nature. His gait is relatively fluid and he is able to run and turn with ease, skip, and perform heel and toe walking.

Using established methods,^3^ whole genome sequencing using a broad neurology panel of 3447 genes revealed a novel heterozygous stop‐loss variant in *STXBP1* [NM_001032221.6] c.1783 T > C, p.(*595Glnext*67), predicting abolition of the stop codon and addition of 67 amino acid residues at the C‐terminus. The variant, confirmed by Sanger sequencing, is absent from gnomAD. Segregation studies were not possible due to familial estrangement. No other pathogenic variants were identified. MRI brain and spine scans age 1, 3, and 5 years showed only mild hypomyelination at 3 years. Prior genetic testing for microarray CGH, fragile X single‐gene testing, and clinical exome analysis of panels for ataxia, dystonia and hyperkinesia were negative (N.B. *STXBP1* was not included in these gene panels), and neurometabolic investigations on blood, urine, and CSF were non‐diagnostic (Supplementary Information). EEG showed no epileptiform discharges.

Experimental animal models including expression of human disease variants in mice suggest that *STXBP1*‐encephalopathy is related to haploinsufficiency rather than a dominant‐negative mechanism.^4^ Stop‐loss mutations are an infrequent but recognized mechanism of genetic disease, not previously reported in *STXBP1*.^5^ The downstream consequences of the stop‐loss variant are unclear, but may include putative loss‐of‐function effects impacting protein folding, subcellular location or substrate binding due to protein elongation. We propose this variant as the likely cause of disease in this patient due to phenotypic overlap, lack of alternative cause on detailed genetic analysis, and haploinsufficiency intolerance of the gene. However, population databases indicate that stop‐loss changes are less selected against than other nonsense variants.^6^ Segregation analysis was not possible, but given the expanding spectrum of *STXBP1*‐related disease, it can be speculated that one of the proband's parents may also harbor this same variant, which may explain some or all of their difficulties.

Although *STXBP1*‐related disorder is strongly associated with epilepsy (95%) and severe/profound intellectual disability (88%),^2^, ^7^ our case had no history of seizures and only mild cognitive difficulties. To date, movement disorders without epilepsy are only rarely reported in *STXBP1*‐related disease and all described cases have severe ID: 3 girls with ataxia and tremor^8^; one individual with spasticity and tremor^9^; and one with tremor with myoclonus^10^ (Table 1). A recent description bringing together 534 cases of *STXBP1*‐related disorders using Human Phenotype Ontology terms has revealed a broader phenotype; ataxia and tremor are common (25% and 24% of cohort respectively) and seizures are described in 89%, but the combined lack of epilepsy or severe learning difficulties remains rare.^5^ Our case expands the proposed genotype of *STXBP1‐*related disease and illustrates the emerging milder phenotype without epilepsy or significant intellectual disability; it also highlights the importance of including *STXBP1* in movement disorder gene panels for diagnostic next‐generation sequencing analysis.

**TABLE 1:** Summary of reported *STXBP1*‐disorder cases without epilepsy

	This report	Stamberger 2016 (P15)	Stamberger 2016 (P29)	Stamberger 2016 (P44)	Stamberger 2016 (P45)	Gburek‐Augustat 2016 (PI)	Gburek‐Augustat 2016 (PII)	Gburek‐Augustat 2016 (PIII)	Banne 2020	Rauch 2012	Rauch 2012	Hamdan 2011	Kim 2021
Mutation/ inheritance pattern	c.1783 T > C, p.(*595Glnext*67)	c.364C > T; p.Arg122* de novo	c.17 T > C; p.Leu6Pro de novo	c.703C > G; p.Arg235Gly de novo	c.795‐1G > A de novo	c.247‐1delG, p.? de novo	c.795‐1G > A, p.? de novo	c.1162C > T, p.Arg388* de novo	c.116_118dup, p.Arg39dup de novo	c.301G > C, p.(Ala101Pro)	c.247‐1del, p.? unknown	c.1206delT, p.(Tyr402*) de novo	c.1439C > T unknown
Sex, age at report	**male, 9y**	female, 5y	female, 7y	male, 3y	female, 11y	female, 6y	female, 11y	female, 11y	female, 5y	female,?	female,?	male, 21y	female, 8y
Movement disorder	**resting and intention tremor, dystonic distal limb posturing, ataxia, head tremor as infant**	truncal tremor, intentional tremor, truncal ataxia	dyskinesia limbs and trunk and choreatiform movements triggered by excitement/action	intention tremor, jerky movements, choreatic or even ballistic movements, stereotypies, ataxia	tremor, ataxia, hypotonia	truncal hypotonia, limb hypertonia, ataxia, head tremor as newborn, intention tremor of the hands since infancy	generalized hypotonia, ataxia, head tremor as infant, intention tremor since infancy	hypotonia, ataxia, intention tremor of hands	tremor and excessive startle from infancy, eye‐rolling at 2.5y, hypotonia and spasticity at 5y	NA	ataxia, tremor	fine tremor	head tremor, dyskinesia, bruxism, hand stereotypies
Development	**delayed; walked at 4y, first words 2y**	GDD from early age: walked at 2y 8m, first words 3y	8m motor delay, hypotonia, GDD from 15m	GDD from 6 m	GDD from infancy	GDD from 6m walked at 3y	GDD from 4m walked at 3.5y	GDD from 7m walked at 2.5y	GDD from infancy	GDD from infancy	GDD	GDD walked at 2y	GDD regression
Degree of ID	**none‐mild**	severe	severe	severe‐profound	severe	severe	severe	moderate	severe	moderate–severe	moderate–severe	severe	severe‐profound
EEG summary	**normal**	normal	normal until age 5y when diffuse background slowing was noticed	normal EEGs at ages 14m and 32m	NA	no epileptiform discharges but a little slowing (18m, 2y and 7y)	no epileptiform discharges but a little slowing (3y, 9y, and 12y)	normal (18m)	normal	NA	3–5 s theta waves (amplitude modulation)	intermittent left temporal slowing at 21y	diffuse background slowing
Gait and speech at last assessment	**can walk and run normal speech**	walks though unsteady limited speech	wheelchair‐bound no speech	cannot walk, minimal or no receptive speech	ataxic gait no speech	can walk limited speech	can walk limited speech	can walk limited speech	help with all everyday tasks	NA	NA	can walk single word speech	cannot walk no speech
Behavioral difficulties	**ASD, ADHD**	episodes of irritability and acting out	no	ASD features	no	aggressive behavior	aggressive behavior	aggressive behavior	NA	NA	NA	ADHD	NA
Neuro‐imaging	**MRI: delayed myelination (3y), normal (2y and 5y)**	MRI normal (2y)	MRI: delayed myelination (4y)	MRI: WM lesions, thinning of CC, mild atrophy (15m and 32m)	NA	MRI normal (14m)	MRI normal (8m and 2y)	MRI normal (18m)	MRI normal	MRI: fronto‐temporal atrophy (4y)	MRI: frontal/parietal atrophy	CT normal (4y)	MRI normal

ADHD – attention deficit hyperactivity disorder, ASD – autistic spectrum disorder, CC – corpus callosum, CT – computed tomography, EEG – electroencephalogram, GDD – global developmental delay, ID – intellectual disability, MRI – magnetic resonance imaging, NA – not available, WM – white matter.

## Author Roles

(1) Research project: A. Conception, B. Organization, C. Execution; (2) Data Analysis: A. Design, B. Execution, C. Review and Critique; (3) Manuscript Preparation: A. Writing of the first draft, B. Review and Critique. All authors contributed to the writing of this letter and reviewed the final version.

RS: 1B, 1C, 2C, 3A, 3B

DS: 1B, 1C, 2B, 3B

KB: 1B, 1C, 2B, 3B

PP: 2B, 3B

EW: 2B, 3B

MK: 1A, 1B, 1C, 2A, 2C, 3B

## Disclosures

### Funding Sources and Conflicts of Interest

The authors declare they have no conflict of interest relating to this manuscript. This study was funded by an NIHR Professorship (MAK, DS, RS), The Sir Jules Thorn Biomedical Award for Research (MAK, KB) and Rosetrees Trust (MAK, KB). The views expressed are those of the authors and not necessarily those of the NIHR.

### Financial Disclosures for the Previous 12 Months

RS's salary is supported by grants from the NIHR, Great Ormond Hospital Children's Charity, and LifeArc; DS's salary is supported by grant from the NIHR; KB's salary is supported by grant from the NIHR, with research supported by grants from NIHR, Sir Jules Thorne and Rosetrees trust; MAK's salary is supported by grant from the NIHR, with research supported by grants from NIHR, Sir Jules Thorne, Rosetrees trust, Great Ormond Hospital Children's Charity, and LifeArc; PP and EW have nothing to disclose.

### Ethical Compliance Statement

The study was approved by the National Research Ethics Service in the United Kingdom (National Research Ethics Service Committee: London‐Bloomsbury, REC reference: 13/LO/0168), and performed in accordance with the Declaration of Helsinki. The family have provided written consent for analysis and publication, including the acquisition, editing and publication of the video. We confirm that we have read the Journal's position on issues involved in ethical publication and affirm that this work is consistent with those guidelines.

## Supporting information


**Appendix S1 Supplementary information.** Includes details of the non‐diagnostic investigations.
