# Supplementary material for: STXBP1 Stop‐Loss Mutation Associated with Complex Early Onset Movement Disorder without Epilepsy
Source: Mov Disord Clin Pract. 2022 Jul 23;9(6):837–40. doi: 10.1002/mdc3.13509 (PMC9346254; doi:10.1002/mdc3.13509)
Supplement: Supplementary file 1 — Appendix S1 Supplementary information. Includes details of the non‐diagnostic investigations. [file MDC3-9-837-s001.docx]

## Supplementary information

Supplementary information includes details of the non-diagnostic investigations:

### Non-diagnostic neurometabolic investigations:

MRI brain and spine

Blood: amino acids, immunoglobulins, acylcarnitines, lactate, glucose, very long chain fatty acids, blood film (for vacuolated lymphocytes), FBC, white cell enzymes (note moderately low arylsulphatase A – 12.7nmol/hr/mg, normal 22-103, suggests heterozygous state), caeruloplasmin, copper, folate, thyroid function tests, alphafetoprotein, biotinidase, transferrin electrophoresis, porphyria (erythrocyte hydroxymethybilane synthase), cholesterol, ammonia, urate, creatine kinase, LFTs, beta HCG

CSF: microscopy, culture, protein, glucose, lactate, neurotransmitters including pterins, oligoclonal bands, amino acids, microscopy

Urine: organic acids, amino acids

### Non-diagnostic genetic investigations:

Microarray CGH analysis – no clinically significant copy number changes, resolution 0.2Mb

Fragile X testing – FMR1 CGG repeat analysis – 28 repeats, in normal range

Clinical exome – virtual panel of genes associated with ataxia, dystonia and hyperkinesia (134 genes)
